# Gender inequality in workloads explained by operational sex ratio

**DOI:** 10.1016/j.isci.2024.110063

**Published:** 2024-05-21

**Authors:** Yuan Chen, Erhao Ge, Liqiong Zhou, Juan Du, Ruth Mace

**Affiliations:** 1 Department of Anthropology, University College London, 14 Taviton Street, WC1H 0BW London, UK; 2 State Key Laboratory of Grassland and Agro-Ecosystems, College of Ecology, Lanzhou University, 222 Tianshui South Road, Lanzhou, Gansu Province 730000, P.R. China; 3 Institute for Advanced Study in Toulouse, Université de Toulouse 1 Capitole, France

**Keywords:** Gender, Social sciences, Anthropology

## Abstract

Ecological differences between human populations can affect the relative strength of sexual selection, and hence drive gender inequality. Here, we exploit the cultural diversity of southwestern China, where some village sex ratios are female-biased, in part due to a proportion of males entering monastic celibacy, to evaluate the role of sex ratio on the sexual division of labor. We used a detachable activity tracker to measure workload by step counts in both sexes among 561 individuals in 55 villages in six different areas. We show that a lower sex ratio and a higher prevalence of monasticism are associated with higher women’s workloads and reduced men’s workloads in the non-celibate population. As the operational sex ratio increases, gender inequality diminishes. This study offers valuable insights into the origins of gender disparities by examining the role of sex ratio on the sexual division of labor.

## Highlights

• Monastic celibacy generates low sex ratios that increase female and reduce male workload
• We show that gender inequality diminished as the operational sex ratio increased

## Graphical abstract

**Figure:**
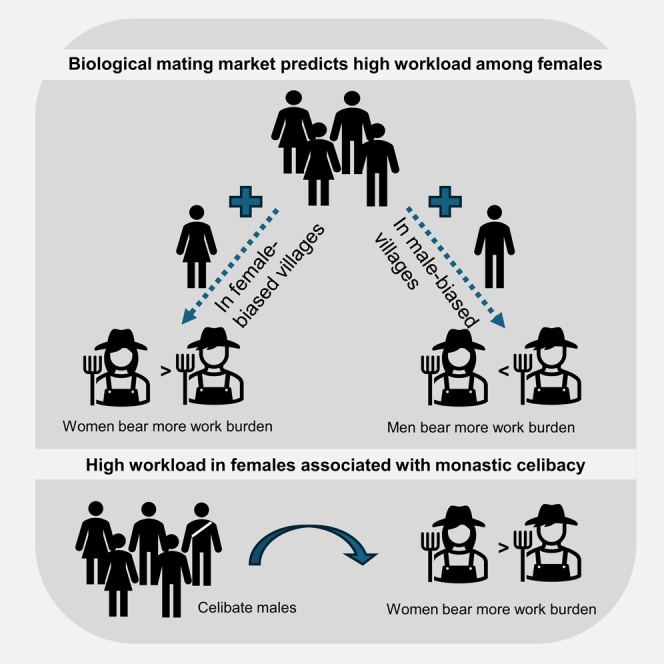

## Introduction

In human populations sex ratio at reproductive age has been shown to act on an individual’s fitness and influence mating and reproductive strategies,^1^^,^^2^^,^^3^^,^^4^^,^^5^^,^^6^ but there has been little research on whether the sex ratio affects workloads. An empirical indicator of the intensity of sexual selection, the operational sex ratio (OSR), can vary due to the sex ratio at birth, and any sex-biases in mortality or migration, and, in humans, celibacy.^7^^,^^8^^,^^9^^,^^10^ This could influence the probability of finding a mate, or remarrying after divorce, and hence the capacity to bargain over resources within households and populations.^11^^,^^12^^,^^13^^,^^14^ Whether this can generate sex inequalities in workloads remains under-researched, particularly in humans.^15^^,^^16^

Adaptive theory generally assumes two sexes bargain in a biological mating market, with one sex being choosy, and the other competing for mates.^12^^,^^17^ Under equal power dynamics, both partners can arrive at a stable equilibrium of contributions to a household. Under unequal power dynamics, affected by skewed sex ratios, an unstable equilibrium, when one of the partners can contribute everything, and the other contributes nothing predicts a downward spiral of divorce-threatened bargaining.^18^ Under such a biological bargaining framework, complementary investment by both partners in a family is essential to understand human energy allocation and trade-offs between mating effort and parenting.^18^^,^^19^^,^^20^^,^^21^

Across much of China, in predominantly Han-dominated areas there is a high sex ratio on account of strong son preference and deep-rooted Confucian values, as well as patriarchal family systems.^22^^,^^23^^,^^24^ But not all local areas have male-biased sex ratios. Historically, in Tibetan areas, up to one-third of males served as celibate monks, leaving home to live in monasteries, where they are out of the mating pool.^25^^,^^26^

Monasticism is a prevalent practice found worldwide, particularly within Buddhism, Hinduism, and Christianity.^27^ Typically, monastic celibacy is observed among males, but in certain ethnographic contexts it can also be seen among women.^28^ Some ethnographers have suggested male monasticism may increase women’s workload.^29^ The decision to forgo reproductive potential poses an evolutionary puzzle.^30^ Mathematical models, supported by empirical research, suggest that parents may favor the adoption of lifelong celibacy for a son to relax competition among siblings.^31^ However, individuals practicing religious monasticism, thereby removing themselves from the mating pool within villages, generate a local operational sex ratio (OSR) that is skewed toward females, creating an unfavorable marriage market for women.

Western China has a diversity of ethnic groups, generating a range of sex ratios within broadly the same overall political and ecological setting. A high proportion of males and very few females joined monasteries to live as celibates in some areas.^31^^,^^32^^,^^33^ Monasteries are somewhat isolated communities, usually outside villages up on hillsides, and can be quite large complexes in some cases. Monks from our study area generally inhabited monasteries, but individual monks are still registered as being part of their natal family. Monks provide religious services to their natal families and the wider community in nearby villages (for which they are paid) but live in monasteries away from the village community; and other than providing religious services, they were never observed working outside the monastery on farms or herding. The monasteries rely substantially on donations of food and money from local villagers. The practice of lifelong male celibates entering Tibetan Buddhist monasteries, at high levels in some of these locations and not in others, caused the sex ratio to vary from village to village. Thus, variation in the number of males leaving to join monasteries offers a quasi-natural experiment in generating biased sex ratios in the villages from which these monks originated.

**Figure 1:**
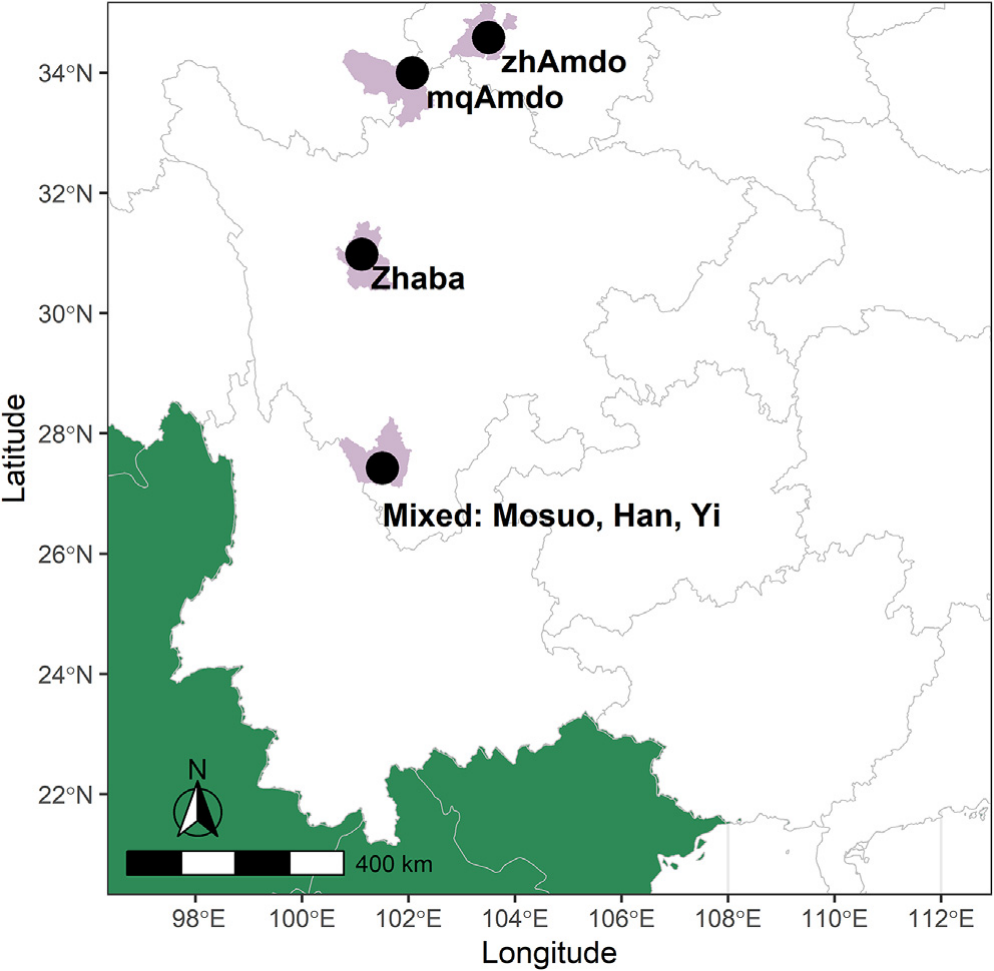
A map of the study sites See also Figure S1.

We conducted a population census across 55 villages from six ethnically different populations (Figure 1), four of which are Tibetan Buddhist cultures. Although these groups share much in common politically and ecologically, including religious beliefs, they differ in their systems of inheritance and descent, and in marriage norms (see more details in STAR Methods). For instance, lineage membership and transmission of resources occur through the female line rather than male line among the Mosuo and Zhaba group; the marriage bond is looser and postmarital residence is mainly duolocal (where husband and wife live apart). Subsistence also differs (see Table 1 and STAR Methods): matrilineal Zhaba group and matrilineal Mosuo, and two patrilineal groups of Han and Yi practice subsistence agriculture and the other two patrilineal Amdo groups keep much more livestock. We studied a sample of the population with activity trackers with individuals’ daily step counts as proxy of workload; and we collected their demographic characteristics, to examine the effect of the village sex ratio on the relative workload of each sex and the specific role of monastic male celibacy. The sample of individuals wearing activity trackers was of villagers and did not include any monks. Given the cultural differences among study groups, we include ethnic groups as random effects to broadly control for cultural differences.

**Table 1:** Average village size, the number of sampled villages, inheritance system, sample size by ethnic groups (*n* = 561 people)

Site	Ethnic group	Village groups	Average village size (population)	Inheritance system	Primary subsistence	Sample size Female (total)
*Lugu Lake*	*Mosuo*	12	211 ± 159	Matrilineal	Mainly farming	66(130)
	*Han*	6	96.5 ± 111	Patrilineal	Mainly farming	26(51)
	*Yi*	4	225 ± 55.7	Patrilineal	Agro-pastoralism	50(95)
*Zhuoni*	*Amdo*	10	169 ± 62	Patrilineal	Agro-pastoralism	44(62)
*Zhaba*	*Zhaba*	16	181 ± 75	Matrilineal	Agro-pastoralism	77(159)
*Maqu*	*Amdo*	7	83.9 ± 63.1	Patrilineal	Pastoralism	31(64)

Adapted from Chen 2023. Mosuo, Han, and Yi are interviewed in the same field site of Lugu Lake. See also Figure S1.

This is the first large-scale study examining the workload differences between the sexes under different operational sex ratios and uses accelerometer technology to document the overall physical activity to test these sex differences. It has the powerful advantage of comprising multiple groups with different social systems and sex ratios within a single country.

Drawing upon the concept of divorce-threatened bargaining within the framework of biological mating market theory, we put forth the following hypotheses:

(1) In villages where one sex is more common, that sex is expected to have a greater workload.
(2) A higher rate of male celibates is predicted to increase women’s workload, while non-celibate males will experience reduced workloads as a result.

All villages were selected at random, and all households were interviewed in those sampled villages to get an accurate estimation of OSR (Figure S1). Villages, as small administrative units where community members interact, varied in size from a few households to several dozens of households, accommodating approximately 160 residents within a single community. Each ethnic group has its own terminology for “village”, e.g., in mqAmdo, the term “village” often refers to a joint-household group (联户组)*,* where herders reside in relatively centralized households, whereas in other groups, it is commonly referred to as a natural village (自然村) (see Table 1 for mean village population size per ethnic group). During the census survey, an average of 98 adults per village were interviewed, and the demographic data revealed a slight male-biased adult sex ratio on average, but much variation. We used the proportion of males among adults aged 14–50 as a measure of the adult sex ratio (ASR).^16^ The operational sex ratio (OSR) is not equivalent to the adult sex ratio (ASR) since individuals practicing lifelong celibacy are excluded from the operational sex ratio. In the Yi and Han ethnic groups, no religious celibacy was observed. Consequently, the OSR value corresponds to the ASR value in villages of these two ethnic groups.

We used activity trackers to assess the overall workload of everyone who volunteered for the study.^10^ All those in the study are adult villagers (not monks). In this paper, we are using the term “workload” to refer to step count data, which we have shown to be correlated with workload by other measures, for instance, a stability validation in a laboratory setting, an experimental validation in real-life scenarios, and permutation models (see details in STAR Methods and in the study by Chen et al.^10^). Our aim is to measure changes in sex-specific work rates with sex ratio (to test our hypotheses) rather than to measure absolute energy consumption.

## Results

### Results of prediction 1: Biological mating market predicts high workload among females

**Figure 2:**
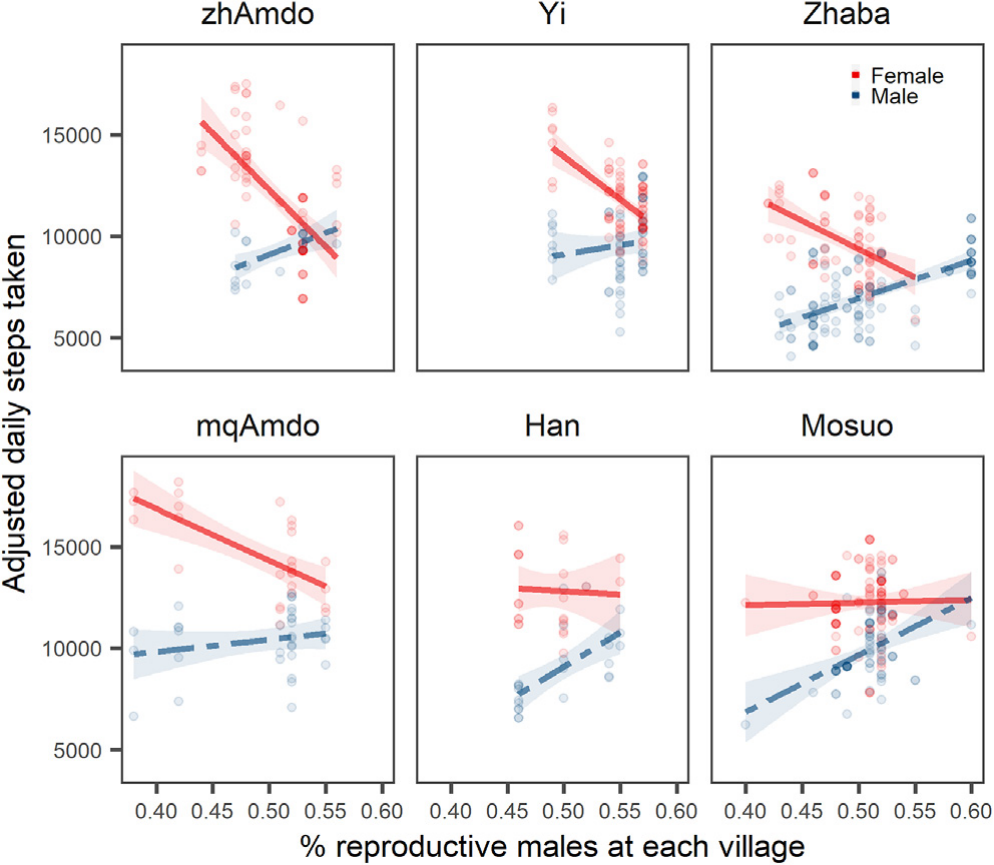
Effects of sex, and operational sex ratio (age between 14 and 50 years and monk removed) on an individual’s daily workload by ethnic group predicted by the OSR model (with age cohorts adjusted) The *x*-axis depicts the proportion of reproductive males aged between 14 and 50 with monks excluded in each village (OSR), while the y axis represents the predicted daily workload of individuals measured in daily step counts. Shaded regions are 95% confidence intervals for the spline value. See also Figure S2.

On the basis of the validated overall daily workload, and individual-level and village-level covariates, a generalized linear multilevel mixed model (GLMM) framework was used to examine the impact of the sex ratio on participants’ daily workload. Univariate model estimates indicate males had significantly lower step counts than females, as we have shown previously.^10^ The multivariate mixed model revealed that women undertook an overall greater workload than men across populations, even after controlling for age cohort. However, as OSR_V_ (the proportion of reproductive males aged between 14 and 50 with monks excluded in each village) increases, women’s work burden gradually declines (Figure 2).

These results support the hypothesis that the operational adult sex ratio is positively correlated with male workload, in accordance with biological mating market theory. We found significant differences in workload in male-biased and female-biased villages for men (step counts for men in male-biased villages: mean = 9602.49, s.d. = 1809.12, *n* = 186; for men in female-biased villages: mean = 7605.81, s.d. = 1891.41, *n* = 110; Linear multilevel models). We also found significant differences in workload for women (women under male-biased sex ratios: mean = 11442.46, s.d. = 2126.89, *n* = 227; women under female-biased sex ratios: mean = 12688.51, s.d. = 2491.20, *n* = 117; Linear multilevel models). A negative correlation was also found between the number of reproductive-age men and the overall average daily workload of all individuals, irrespective of their sex (Table S1). We also modeled the relationship between the workloads of single men (*n* = 66 observations) and single women (*n* = 31 observations), controlling for age cohort. The data among single individuals also supported our hypothesis that single women work much more than single men under female-biased contexts, and vice versa (Figure S2). Our result indicated that a greater number of reproductive-age men reduces workload, implying that the overall decline in everyone’s workload may be driven by the shortage of women laborers, or that monks are a net cost.

### Results of prediction 2: High workload in females associated with monastic celibacy

Operational sex ratio varies by ethnic group and by village. Our data show that 0.9% of the total Mosuo population are monks (*N* = 2737 residents of both sexes), 4.8% of the Zhaba population (*N* = 2896 residents), 2% of the mqAmdo population is lower (*N* = 587 residents) and 4.5% in the zhAmdo population (*N* = 1688 residents). But the % of monks in each village varied considerably. We observed a village maximum of 20.49% of male adults (aged between 14 and 50) being monks, with a considerably lower mean and high variance (Mean = 3.91%, Q1 = 0%, Q3 = 6%, *N* = 55 villages) (Figure S3). There were no monks among the Yi or the Han (Figure S3), hence ASR and OSR are the same value in the Han and Yi sample villages.

**Figure 3:**
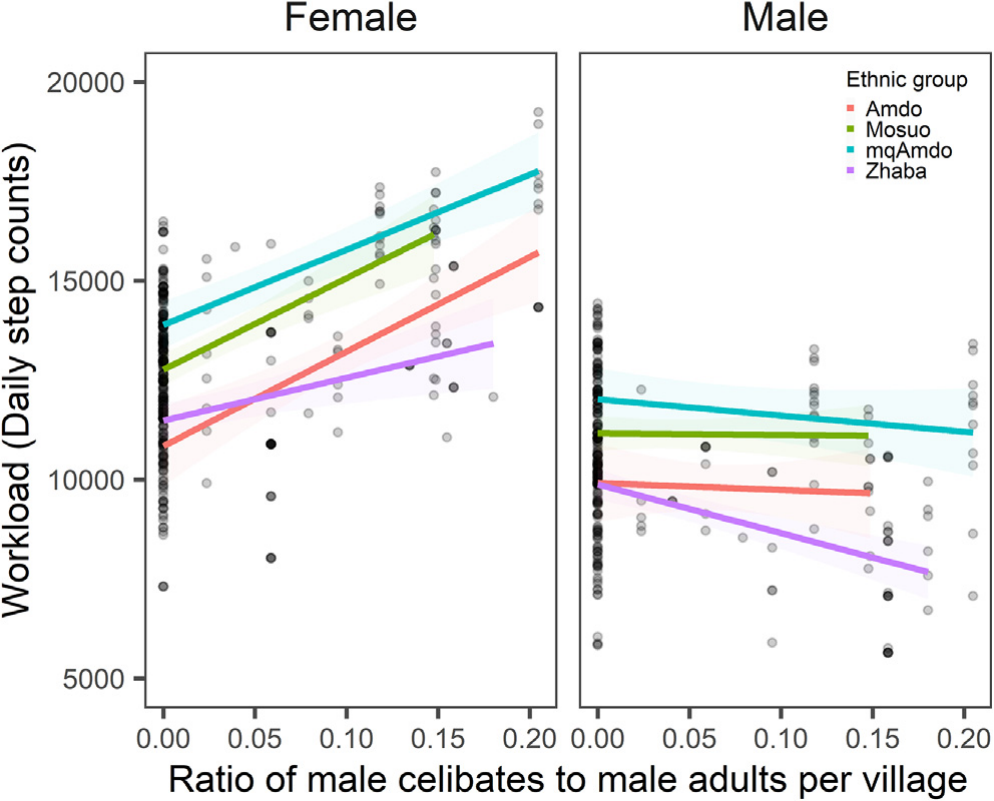
Effects of male celibates on non-celibates’ workload by sex and ethnic group The *y* axis indicates the daily step counts (workload) estimated by the multilevel model (see Figure S4 for model details). The *x* axis was estimated by the ratio of male celibates to all male adults (ages 14–50). The shaded region indicates a 95% confidence interval. The frequency of male celibates of Yi and Han (not shown in Figure 3) was measured as zero. Orange (zhAmdo), Green (Mosuo), Blue (mqAmdo), Purple (Zhaba). See also Figures S3 and S4.

We examine the direct relationship between the prevalence of monastic celibacy in a village (that is the proportion of males born in the village who leave to become monks) and the individual workload of non-celibates of both sexes. Our analysis reveals a significant positive association between the proportion of males becoming monks in a village and the daily workload of all residents, after accounting for socioeconomic characteristics (Beta = 17158.13, SD = 7134.09, 95% CI [3266.62, 31049.64], *p* = 0.016; Linear multilevel model; see Figure S4). This suggests that an increase in the number becoming monks may contribute to a higher overall work burden, potentially due to the financial support provided to the monastery by their families and other villagers. Importantly, a higher percentage becoming monks among total male adults is associated with a substantial decrease in local (non-monk) men’s work effort (Beta = −24437.96, SD = 9383, 95% CI [-42708, −6167], *p* = 0.0094; Linear multilevel model; Figure S4). The impact of monastic celibacy on women’s work burden is more pronounced than its effect on men’s workload (Figure 3). Our data indicate that each additional one-tenth of an adult male becoming monks will raise women’s workload by 1715.81 step counts and decrease men’s workload by 727.98 step counts (Figure 3 & Figure S4).

Our findings suggest that the practice of male monastic celibacy contributes to the higher workloads experienced by women. In line with our hypothesis, the presence of celibate males drops the local operational sex ratio, leaving women in a disadvantageous position in the marriage market, while non-celibate males gain greater bargaining power in intersexual interactions.

### Role of monastic celibacy in driving sex inequality

Sex inequality at the village level is further explored using an estimate of the “gender step gap” in labor effort, calculated as the difference between females’ work effort and males’ work effort within each village. The OSR model adjusted for individual characteristics was used to determine the average labor effort for males and females, respectively. We compared several models to investigate whether ethnic groups as a random effect are having any other effects or whether the operational sex ratio can explain all the variance in work effort disparity by sexes.

Our analyses, as presented in Table S2, revealed that the global model accounted for a greater proportion of the variance in the gender step gap. Using a multilevel approach, we found a significant negative association between the gender step gap and OSR (Beta = −35551.79, 95%CI [-45693.06, −25410.52], GLMM, correlation matrix, R = −0.911). This indicates that as the operational sex ratio becomes more male-biased, the difference in step counts between the sexes diminishes, and the effects of ethnic differences are primarily explained by the sex ratio.

In villages with a female-biased OSRs ranging from 0.38 to 0.50, the mean step gap between sexes was 5462.21 ± 1333.17, while in villages with an OSR ranging from 0.50 to 0.60 (i.e., male-biased), the mean step difference was 1804.35 ± 1105.62. Compared to other ethnic cultures, we observed that the matrilineal Mosuo exhibited greater gender equality in workload despite having a female-biased OSR (Figure S5).

Following this, we estimated the sex ratios at the ethnic group level to investigate how the dynamic biological mating market operates on an ethnic basis. A full model was constructed with different sex ratios (ASR_V_ vs. OSR_V_, and ASR_E_ vs. OSR_E_); after model selection, the OSR_v_ model appeared to be the best-fitting model for predicting workload at this level.

**Figure 4:**
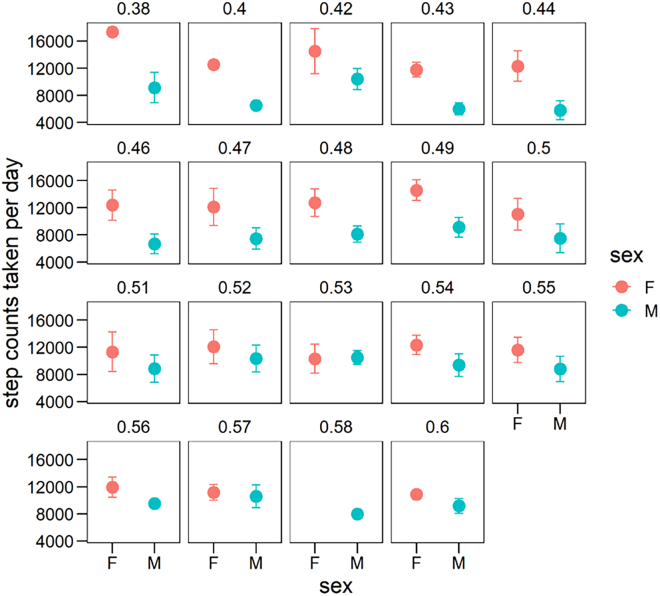
Daily step counts gap taken between sexes varies with sex ratio Red (F) indicates married females; Green (M) indicates married males. Each facet shows the average step count value of a specific adult sex ratio when the decimal points were limited to two, as calculated from multiple villages. See also Figure S5.

Taking these together, it becomes evident that the practice of monastic celibacy plays a significant role in shaping gender inequality in workload. Many young men opting for celibacy leads to a decrease in the operational sex ratio, thereby creating a gender imbalance for those remaining in the village. In settings where females outnumber males, women bear a significantly higher work burden compared to men. For instance, in a scenario with a female-biased operational sex ratio of 0.38, women are predicted to shoulder twice the workload of men (Figure 4). These shifts in the marriage market, originating from the prevalence of male monastic celibacy, have a profound influence on individual social behavior, and are the most important underlying mechanism underlying any differences between ethnic groups.

## Discussion

The origin of gender inequality is of interest within the social and biological sciences as well as in governments and the general public, as it has the capacity to harm health, economic development, and quality of life. Much of the discourse on gender inequality focuses on cultural context, but here we show how population structure can be a driving force for gender disparities in work burden. The practice of male monastic celibacy increases the workload for females, lightening workloads for non-celibate men. Hence it is population structure, affected in part by the prevalence of males leaving the village to become celibate monks that drives a greater sexually selected workload.

We posit that when men possess greater bargaining power in the marriage market, they may have more leisure time at their disposal to allocate toward pursuits such as mating strategies or elevating their social standing. Such endeavors could yield individual benefits rather than shared interests, contributing to the observed discrepancies in workload. Skewed OSRs can result in intense competition for mating opportunities among the more abundant sex, leading to various evolutionary adaptations shaping behavior, morphology, and reproductive tactics. Here, we show that operational sex ratio (the number of available mates) predicts a bargaining power asymmetry between males and females, especially for those of reproductive age. The more labor effort contributed by one sex to the family, the more the other can allocate resources to processes that promote their individual survival, status, or intrasexual competition for resources and mates, or extra-pair mating.^34^ An excess of females could result in cooperation breakdown between parents and even invoke a higher divorce risk.^8^^,^^33^

Male excess may promote male parental effort and hence stabilize the pair-bonds due to restricted future reproductive potential should they leave a partnership.^8^^,^^35^^,^^36^ Alternatively, abundant males may opt for more violence due to intense mate competition if males could gain more by competing rather than cooperating.^37^ A study based on national-level data from the United States found that in male-biased sex ratios contexts, criminal and violent behavior related to the male mating effort was low,^38^ and negatively correlated with violence against women.^3^ Given the high energy demands of human female reproduction, the offer of male labor is a salient factor for women’s reproductive success^39^; male efforts in production, childcare, and income may serve as a strong predictor of child growth, more appealing to females when females are the choosier sex. A meta-analysis of experimental data on brood size manipulations in birds suggested increased workload might be even more harmful to males’ survival than females, albeit brood-size manipulation in this study did not serve as a powerful indicator of parental workload.^40^ A review of human reproduction and health indicated that the mortality of men is greater when their energy consumption is the same as women ^41^^d^. These predict a women’s mate preference for men who contribute significantly to subsistence when the ecological context is male-biased.

While celibacy among males means they are out of the mating and marriage market, they may still be able to enhance their family’ fitness by both reducing sibling competition^32^ and potentially elevating the social standing of their kin.^42^

Only when the operational sex ratio achieves a male bias is there an equal workload between sexes. We have previously shown that females have less bargaining power generally, and both sexes have less bargaining power if they disperse at marriage.^10^ In our current study, we have shown an association between the degree of monastic celibacy in villages and the sex inequality observed in workloads. We discovered females continue to experience some workload cost of celibates even if the operational sex ratio reaches 0.5. This may be because monks continue to receive donations of food and resources from their family to them personally and to the monastery in general (monasteries in these regions do not generally farm at all); so, females may do extra work to support their siblings who are celibate monks living in the local monastery.

Our findings reveal that the highest level of sex inequality in workloads is observed in the mqAmdo population, which has the highest proportion of monks in the reproductive age group. In contrast, the Mosuo, a matrilineal society characterized by a duolocal residence pattern (where married partners live apart), displays the lowest sex inequality in workloads. Women living in female-centered kin-based group households may defect at relatively low cost, even under a highly unfavorable sex ratio, as co-resident females are providing the help to raise offspring anyway.^43^ Together, our quasi-natural experiment approach, utilizing the influence of monastic celibacy, provides insights into population dynamics and its impact on sex inequality in workloads. By considering the interplay between the OSR, monastic celibacy, and social structures such as matrilineal societies, we gain a more comprehensive understanding of the factors influencing bargaining power, sexual selection, and the division of labor between sexes. Conditioning on the practice of monastic celibacy—our quasi-natural experiment approach, suggests that sexual selection favors the hard-working sex when there is sexual bargaining.

### Conclusions and limitations of the study

However, it is important to acknowledge that untangling the intricate relationships between the operational sex ratio (OSR) and various village-level factors, including economic systems, wage labor dynamics, inheritance practices, and sociocultural norms, or an unobserved common cause, e.g., sex-biased mortality,^44^ warrants further investigation. Establishing definitive causal links between the OSR and environmental variables remains a challenge. Therefore, caution must be exercised in interpreting the OSR’s comprehensive influence on village-level characteristics and socioeconomic dynamics. Our study context predominantly encompasses rural populations with limited exposure to urban economies or sedentary jobs, as they rely primarily on subsistence-based livelihoods rooted in natural resource utilization through farming and herding. Consequently, we do not think our results are confounded by an association between the OSR and time allocated to wage labor in these rural and semi-nomadic communities. Our focus is on examining the distribution of physical labor across sexes rather than overall productivity. As a proxy for measuring workload, lower step counts among men indicate a higher engagement in leisure activities or sleeping.^10^ Additionally, it is worth noting that women’s work is often performed in the rain, whereas certain tasks assigned to men may be limited under such conditions.

To conclude, while there can be many causes of biased sex ratio, here we identify monastic celibacy as a factor that drives down OSR because it removes many adult men from the reproductive pool in some areas and not in others. In an ecological setting with female-biased operational sex ratios, driven by men leaving to become celibate monks, women work harder. While individual monks may not gain advantages, this system may have reinforced gender inequality in the general population in favor of non-celibate men and contributed to the establishment of the patriarchal norms that prevail.

## STAR★Methods

### Key resources table

**Table:** 

REAGENT or RESOURCE	SOURCE	IDENTIFIER
**Deposited data**		

Activity data and Demographic data	This paper	UCL Research Data Repository: https://doi.org/10.5522/04/25388287

**Software and algorithms**		

Codes and outputs of GLMM analyses	https://github.com/YUANDALAO/SEX-RATIO-PROJECT.git	Zenodo: https://doi.org/10.5281/zenodo.10806908

### Resource availability

#### Lead contact

For additional information and resource inquiries, please direct your requests to the lead contact, Ruth Mace (r.mace@ucl.ac.uk).

#### Materials availability

This study did not produce any novel reagents or materials.

#### Data and code availability

• Any personal data that could be identifiable is not openly available, in line with ethics protocols. The anonymized workload data is stored in the UCL Research Data Repository. The DOI is listed in the key resources table.
• All original code has been deposited at GitHub https://github.com/YUANDALAO/SEX-RATIO-PROJECT.git and is publicly available as of the date of publication. DOIs are listed in the key resources table.
• Any additional information required to reanalyze the data reported in this paper is available from the lead contact upon request.

### Experimental model and participant details

#### Study populations

We worked in 55 villages across six areas with different ethnic groups: the *Mosuo*, the *Yi*, the *Han*, and the *Zhaba* in Sichuan Province, and two different populations of Amdo Tibetans (*zhAmdo* and *mqAmdo*) in Gansu Province (Figure S1). The *Han* and *Yi* do not practice Tibetan Buddhism. These populations in Southwest China cohabit under the same political context – monogamy is the only legal and the most prevalent form of marriage, with different social system and residence patterns coexisting.

##### Ethnographic context

The Mosuo, Han, Yi, zhAmdo, mqAmdo, and Zhaba are diverse populations residing in different regions of China. The Mosuo, a matrilineal group, with a traditionally female head of the family, primarily inhabit the Lugu Lake area in Sichuan. The families of Mosuo people live in large matrilineal households of three generations of brothers and sisters, and the matrilineal offspring, and the family farm communally.^43^ The Yi and the Han have patrilineal descent (goods and titles inherited along the male line) and do not practice Tibetan Buddhism. The Yi, in the Lugu Lake area, is called ‘*Nuoso*’, while in other places of China, most are called ‘*Nisuo*’, marriage between different separate Yi groups is forbidden and limited by the social hierarchy, as well as marrying other ethnic groups.^45^ In Yi families, the principle of Father-Son Joint Name paternity - offspring’s name is joined after the parents’ name, and patrilineal inheritance of property are practiced, with sons establishing separate households after marriage and only the youngest sons living with their parents after marriage.

Zhaba, located in Sichuan Province, Ganzi Autonomous Prefecture, is another cradle of matrilineal groups in China. Upper - Zhaba habitat is mainly female-oriented, associated with more prevalent walking marriages and female household heads, whereas Lower-Zhaba has more male heads of households, despite that both areas in this community practice matrilineal system. Residents grow barley and potato across the two areas, restricted by natural resources. Spouse-relationship in Zhaba has changed in the past few decades. Like the Mosuo population, the Zhaba also practice ‘walking marriage’. Until recent, with the implementation of family planning policy in 1980s, local people adopted a “New visiting marriage”, where obtaining a marriage certificate after their common offspring was born, however, continued to live apart after pair-bonding.^46^^,^^47^

Tibetans living in Amdo are called *Amdobas* (Tibetan: ཨ༌མདོ; Chinese: 安多); they rely mostly on yak and sheep herding and other forms of agriculture to provide subsistence. The Amdobas who practice farming in conjunction with herding are known as *Rong bas*, whereas nomadic herders who do not rely on farming are called *Brog pa*.^48^ Amdo Tibetans, including zhAmdo and mqAmdo, reside in Gansu Province. Like the *Han* people, with whom there is some intermarriage, these two populations practice a patrilineal system nowadays - that men are the leader of the family and display absolute authority and power in this area. The zhAmdo people are agro-pastoralists who speak the Amdolese Tibetan dialect and are characterized by customs, dress, and religious practices that differentiate them from other Tibetan groups.^49^^,^^50^ The mqAmdo population, predominantly nomadic herders, occupies an area spanning Qinghai, Gansu, and Sichuan provinces, practicing both polygamy and monogamy in their marital systems.

##### Primary economic activities

Among these populations, distinct economic activities shape their way of life. The *Mosuo* community primarily relies on farming, with communal farming practices in large matrilineal households. Domestic labor (except building work) is mostly done by women.^10^ Men are rarely seen in the fields except at planting and harvest time, and men are more regularly involved in income-generating activities associated with a market economy,^48^^,^^51^ but our observation and data confirm that females work harder than males in both domestic and agricultural work.^10^ Residing on low altitude, the Han and Mosuo population are actively engaged in income-generating activities associated with a market economy, as well as agriculture. The Yi community relies on both farming and livestock, together with very limited income-generating activities, restricted by their living ecology on the top of mountain. The Mosuo, Han, and Yi participate in various professions and occupations, reflecting their diverse economic endeavours. Governed by a committee of community and government officials, profits generated by tourism among households are anticipated to be equal. Besides, previous time allocation data found no evidence of sex difference in the participation of tourism.^10^

The Zhaba population relies heavily on agriculture, with highland barley and potato cultivation being their main sources of subsistence. Limited access to education and few craftsmen and artisans contributes to a relatively small number of individuals working in non-agricultural industries. Before the construction of the highway in 1974, this area was totally secluded from modern society. Strike by the modern society and intervention by the external population, more residents gradually adopted from Barter system to Currency Swap (trade mushroom for money). Although mushrooming was not the only way to earn money, it was still commonly seen in local areas, especially in summer season. In early May, local people climbed the highest mountain and sought for the mushroom in the exceedingly early morning to adopt the freshest mushroom to get well paid in the market. It always took all the daytime and they mostly fed on the solid food preparing for this long mushrooming journey, while sometimes they also spent the whole night at the top of that mountain (some families built their family camp there).

Amdo Tibetans, including both zhAmdo and mqAmdo, rely predominantly on yak and sheep herding, along with other forms of agriculture. The zhAmdo practiced semi-agro-pastoralist culture where residents feed on livestock and dairy products and meanwhile rely on the farming lifestyle, while mqAmdo embraces a nomadic herding lifestyle without farming. The mqAmdo population, comprising nomadic yak and sheep herders, greatly sustains their livelihood through livestock herding.

##### Religious beliefs

The religious beliefs among these populations vary significantly. Four of these populations are Tibetan cultures, practicing Tibetan Buddhism. The Amdo Tibetans, both zhAmdo and mqAmdo, have their religious practices intertwined with Tibetan Buddhism, including the Geluk (Yellow Hat) sect. Monastic institutions and celibacy are significant aspects of their religious beliefs. The Maqu population, with its nomadic herding lifestyle, also embraces the Geluk sect. The Zhaba population has a belief system that encompasses both 'Bon' and Tibetan Buddhism, with a focus on the Nyingma or Gelug sects.^52^^,^^53^ Religious practices, rituals, and monastic traditions play an integral role in shaping the Zhaba society.

The Mosuo population adheres to a combination of 'Daba' (Dabaism), 'Bon,' 'Red Sect,' and 'Yellow Sect' of Tibetan Buddhism. Before Buddhism was introduced, - ‘*Bon*’, as the original religion,^54^ was believed by Mongolian and Tibetan people. People also believe in Lamaism the *Red Sect* was centered along the coast (Lugu Lake) – only one red sect monastery is left now in Lugu Lake.^55^ Every family had a hall of prayer, and every man’s family had to send one or two people to serve as celibates. The *Yellow Sect* was introduced into the Lugu Lake area in the late *Ming* Dynasty, but it was strongly resisted by the *Red Sect*, so it had little influence.^55^ Besides, some monks in Lugu lake practice ‘walking marriage’.^55^^,^^56^ These religious practices have a deep-rooted influence on their cultural traditions.

In contrast, the Han population in the studied regions does not practice Tibetan Buddhism and follows a multi-religious belief system. Ancestor worship is commonly observed among the Han and Yi communities, particularly within the patrilineal groups. Prior works suggested that much of the Yi population largely borrowed the main Han culture recently, despite its long history. Regarding religious belief and spirits, they are exclusively distinguished from Han ethnic group, the *Nuoso* believes in everything, while Han now believes in multi-religious, yet believing in ancestors is commonplace in the two patrilineal groups.^57^

#### Data collection

Data was collected by Y.C., E.G., L.Z., and J.D. with local field assistants between 2017-2018 in Southwest China. Ethical clearance was given by UCL Research Ethics Committee (n. 0449/003) and Lanzhou University. Participants were briefed in the local language before giving consent. All participants gave their informed consents.

Demographic data were collected during 2017-2018 from all available village households (N = 1796 households). The head of household, or another responsible adult if the head of the household was not available, gave demographic details of the whole household including whether any individual born in that family had become a monk or nun. In addition, we recruited a sample of the adult residents in the area to wear a triaxial accelerometer for 2-5 days. These participants who involved in the accelerometer session were sampled randomly, with roughly equal numbers of men and women in each ethnic group (see Table 1). All families participating in this study gave consent and a second consent if they agreed to participate in the accelerometer study. After we collected the accelerometer for two to five days, we asked whether they had removed the accelerometer at all in the last 24 hours.

We have shown previously^10^ that dispersal at marriage increases workload - and thus we controlled for that here. Our study locations contained six ethnic groups, including those of patrilineal and matrilineal descent which partially predicts the proportion of females that disperse at marriage. There is variability within each ethnic group and between groups in the proportion of males and females dispersing.^10^ The Yi, Han, and the two different groups of Amdo Tibetans are predominantly patrilineal where females are more likely to disperse, whereas the Mosuo and Zhaba are predominantly matrilineal. These two matrilineal groups (Mosuo and Zhaba) include households with a duolocal residence pattern (neither sex disperses). There are also many variations within groups in residence patterns. All groups include some couples showing patrilocality, matrilocality, or neolocality (both sexes dispersed), but in different proportions.^10^

### Quantification and statistical analysis

#### Validation of activity tracker in the lab and free-living environments

Four participants performed a walking task on the same day, and everyone was asked to wear the two kinds of accelerometers at the same time for twenty minutes. We selected 8 Miband2 and 13 Fitbit Charge2 randomly and separated them into two sets (Set 1: Six Fitbit Charge2 + four Miband2; Set 2: Another seven Fitbit Charge2 and another four Miband2). And subject A and subject B were told to wear the same four Miband2 and seven FitbitCharge2 (Set 1) but in a different period, subject C and subject D wore the same four Miband2 and six Fitbit Charge2 (Set 2) but not the same period. And then we calculated the mean steps over the last twenty mins, standard deviation, variance, and coefficient of variance for each round by both bracelets (referred to as Miband2 and Fitbit Charge2) to measure the stability of each kind of activity monitor (Table S3).

In three ways, we validated our wristband accelerometers (Miband 2) with workload data and check their reliability. To begin, although these activity trackers had been empirically tested before being released to the public, we placed all the activity trackers in the same sack and recorded every single step counts after the same journey, to examine carefully if they performed consistently, and then we eliminated two outlier wristbands that were significantly different from the mean step counts. Secondly, we checked how the wristbands were recording data corresponding to our time-budget allocation data. An example of the raw data from three individuals is also shown in the supplementary material of.^10^ Thirdly, studies of metabolic energy consumption have found that the costs of daily activities vary greatly. Therefore, we recorded 24 categories of activity in total (Table S4), depending on different metabolic equivalent tasks levels (METs), which refers to the ratio of the energy expenditure rate for activity compared to resting energy expenditure, and we classified some subsistence-related activities into L.I. (Low Intensity, energy expenditure below 3 METs), M.I. (Moderate Intensity, energy expenditure between 3 METs and 6 METs), and H.I. (High intensity, energy expenditure above 6 METs) in our analysis abstracted from the Compendium of Physical Activities.^58^

In that, we regressed total step counts (daily step counts are raw activity data derived from Miband 2^10^) as the dependent variable and the previous days’ time allocation data as the predictive variable, using a multivariate permutation regression (Permutation tests, also called randomization or re-randomization tests).^59^ Because step counts are related to height and weight, we controlled for that in subsequent analysis. The results of the generalized linear regression model also revealed that the time reported by participants undertaking subsistence work and gathering was significantly positively correlated with the total counts of steps (Table S5). When individuals spent more time on self-care and leisure, they took fewer steps (Table S5). Overall, these results are consistent with what we expect if the step count is an indicator of the individual’s workload.

#### Statistical analysis

All analyses were conducted in R. We used an alpha level of 0.05 for all statistical tests.

In this study, we use generalized multilevel mixed models (*GLMMs*) with maximum likelihood to investigate how village sex ratios predict the relative workload of both sexes. Covariates used for GLMM models consisted of general demographic variables, including sex, age, body mass index, whether one took the wristband off or not, and whether this ego was during the lactation period or being unwell as control variables. Since we collected data from six ethnic groups, comprising diverse cultures, ethnic groups were also set as random effects in model formulation to control other aspects of cultural difference. We allow the ego’s workload (*W*) of each ethnic group to vary within intercept. Level 1 equation is as follows:

***W***(*x*) = ***β***_0*j*_ + ***β***_1*j*_*X* + *R*_*ij*_

Level 2 equation:

***β***_0*j*_ = ***γ***_00_ + ***γ***_01_*E*_*j*_ + ***μ***_*oj*_, ***μ***_*oj*_ ∼ *N* (0, ***σ***)

In this model formula, Ej corresponded to ethnic groups. We allowed each ethnic group to have diversified baselines, thus,

***β***_0*j*_ = ***γ***_00_ + ***γ***_01_*Z*ℎ*aba* + ***γ***_02_*Mosuo* + ***γ***_03_*Yi* + ***γ***_04_*Han* + ***γ***_05_*mqAmdo*+ ***γ***_06_*z*ℎ*Amdo* + ***μ***_0*j*_

To estimate the good fit of the model as stable as it could be, we employed a sample in each field site as much as possible. To begin, we applied the alternative GLMM method with a Poisson distribution function to compare all-level random effects in a full model more effectively. To model non-Gaussian data, we used a Gaussian model after transforming our non-Gaussian response to make it approximately Gaussian.^60^ Considering zero cannot be log-transformed, we applied a quadratic transformation to the response variable. We predicted sex ratio will have a distinct effect on single sex’s workload, and thus, we added an interaction between sex and sex ratio in the model formulated on assumption that males and females will respond oppositely to the sex bias.

We then dredged our models set, which was generated by limited tailored combinations of models according to the conditions specified,^60^ and all candidate alternative models were eventually compared based on Akaike Interference Criterion (AIC) value, with subsets of the supplied ‘global model’ and eventually the GLMM model with the lowest AIC value was selected as our best-fitting model and its model coefficients were shown as a forest plot in the SIs (Figure S4). Last, as a mediator of the causal effect of monastic celibacy, OSR itself is not included in the model syntax of mediation analysis. This is because, controlling for any mediators, can block the very effect we want to estimate (the total effect of celibates on workload), thus biasing our estimates (usually known as ‘overcontrol bias’).^61^

We chose not to include wage labor and related variables as controlled factors in our analysis. Instead, we focused on investigating the associations between the OSR and the magnitude of workload disparities. Moreover, our previous study did not reveal significant sex differences in wage labor participation across these rural populations, as detailed in Figure S6.
