# Supplementary material for: Gender inequality in workloads explained by operational sex ratio
Source: iScience. 2024 May 21;27(6):110063. doi: 10.1016/j.isci.2024.110063 (PMC11179575; doi:10.1016/j.isci.2024.110063)
Supplement: Document S1. Figures S1–S6 and Tables S1–S5 [file mmc1.pdf]

iScience, Volume *27*

Supplemental information

Gender inequality in workloads explained by operational sex ratio

Yuan Chen, Erhao Ge, Liqiong Zhou, Juan Du, and Ruth Mace

Figure S1.

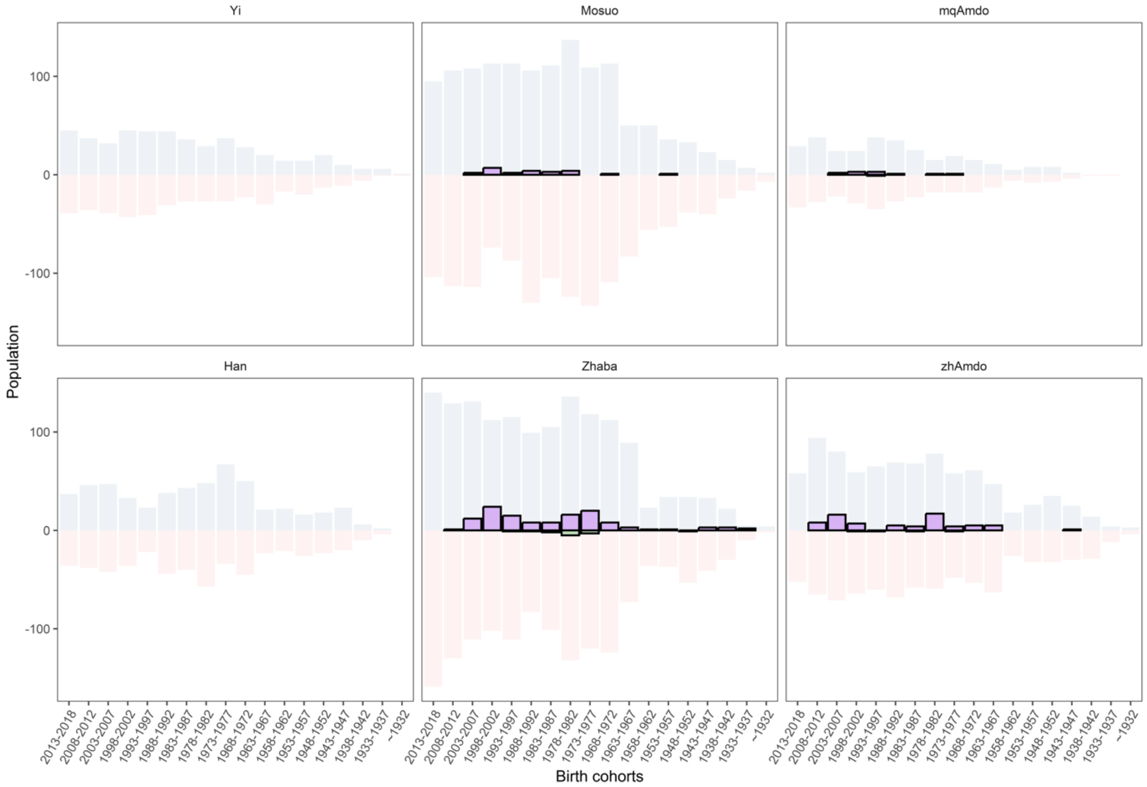

Age distribution of celibates (monks in purple, nuns in green) by ethnic groups, related to Figure 1. The blue indicates males, while red indicates females in pyramid. Amdo, Han, Mosuo, mqAmdo, Yi, and Zhaba are the six ethnic groups included in this study.

Figure S2.

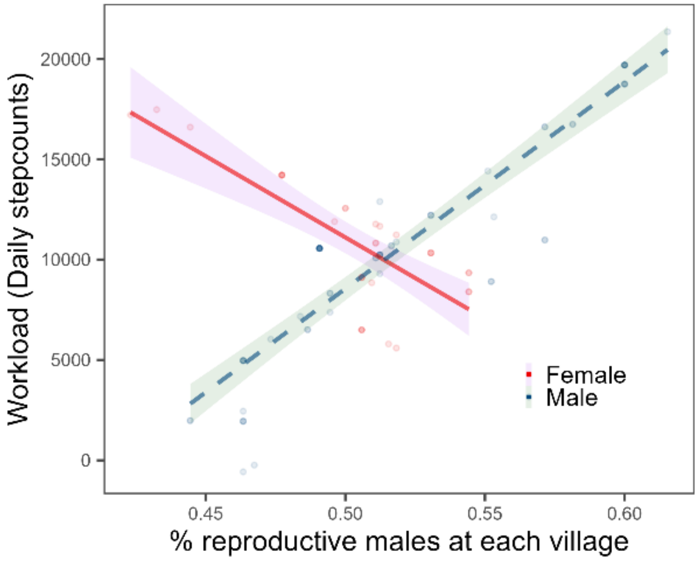

Effects of sex, and operational sex ratio (age between 14-50 yrs. and monk removed) on a single/unpartnered individual's daily workload (with age cohorts adjusted), related to Figure 2. The x-axis depicts the proportion of reproductive males aged between 14-50 with monks excluded in each village (OSR), while the y-axis represents the predicted daily workload of single/unpartnered individuals measured in daily step counts. Shaded regions are 95% confidence intervals for the spline value.

Figure S3.

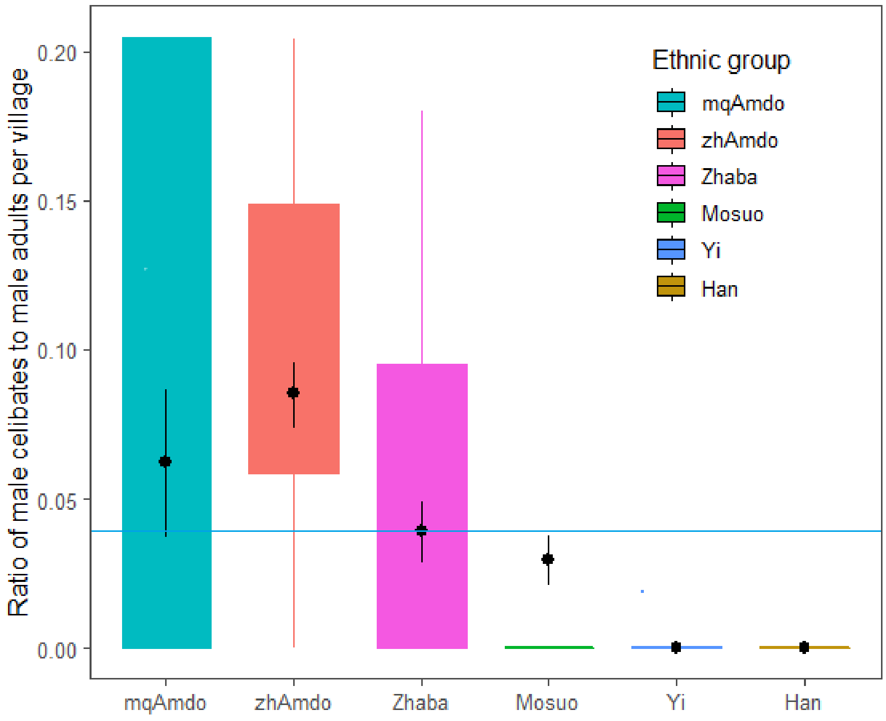

Frequency of male celibates in our study areas, related to Figure 3. ZhAmdo, mqAmdo, Zhaba, Mosuo, Han, and Yi represent six ethnic groups. Orange (zhAmdo), Green (Mosuo), Blue (mqAmdo), Purple (Zhaba). The y-axis represents the ratio of male celibates to all male adults (ages 14-50). The horizontal blue line represents the mean frequency of male celibates (3.91%) across all villages. The black dot inside boxplots indicates a mean frequency value and standard deviation of male celibates among villages within each ethnic group.

Figure S4.

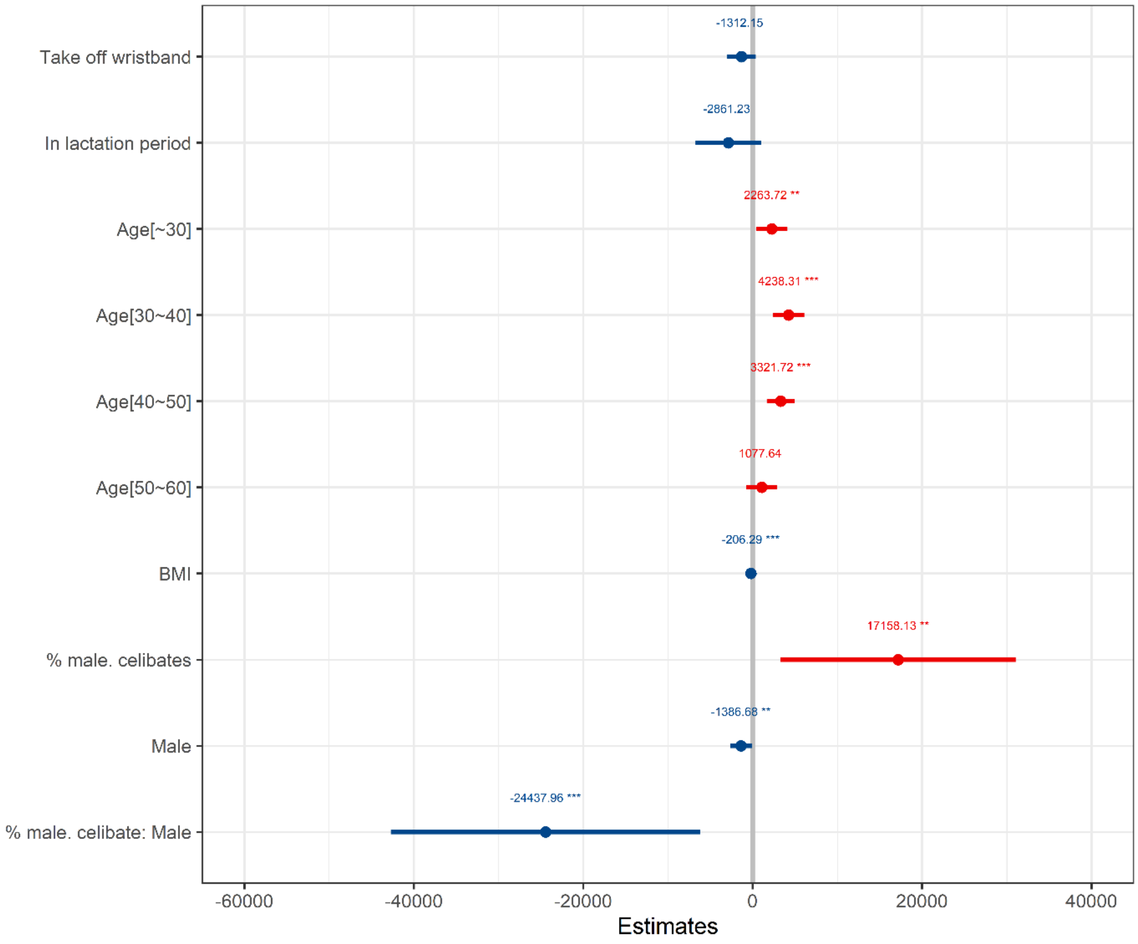

Multilevel model parameters of predicting steps by the percentage of male celibates, related to Figure 3 and STAR Methods. The ethnic group is set as a random effect.

Figure S5.

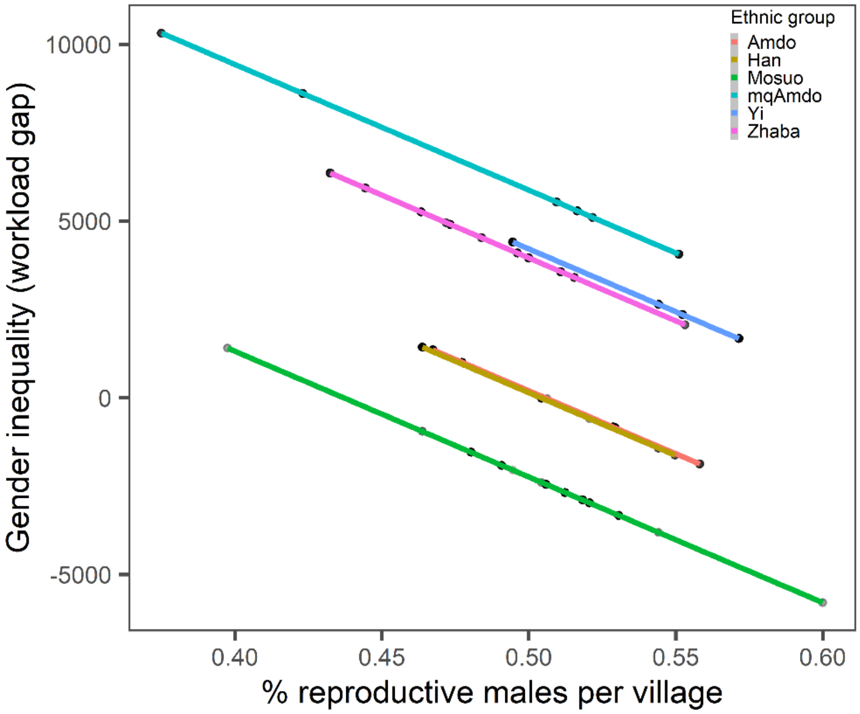

Association between gender gap in workload and operational sex ratio (OSR), related to Figure 4. Amdo, Han, Mosuo, mqAmdo, Yi, and Zhaba are the six ethnic groups included in this study. Dots indicate villages. Lines are fitted from the best-fitting multilevel model.

9

Figure S6.

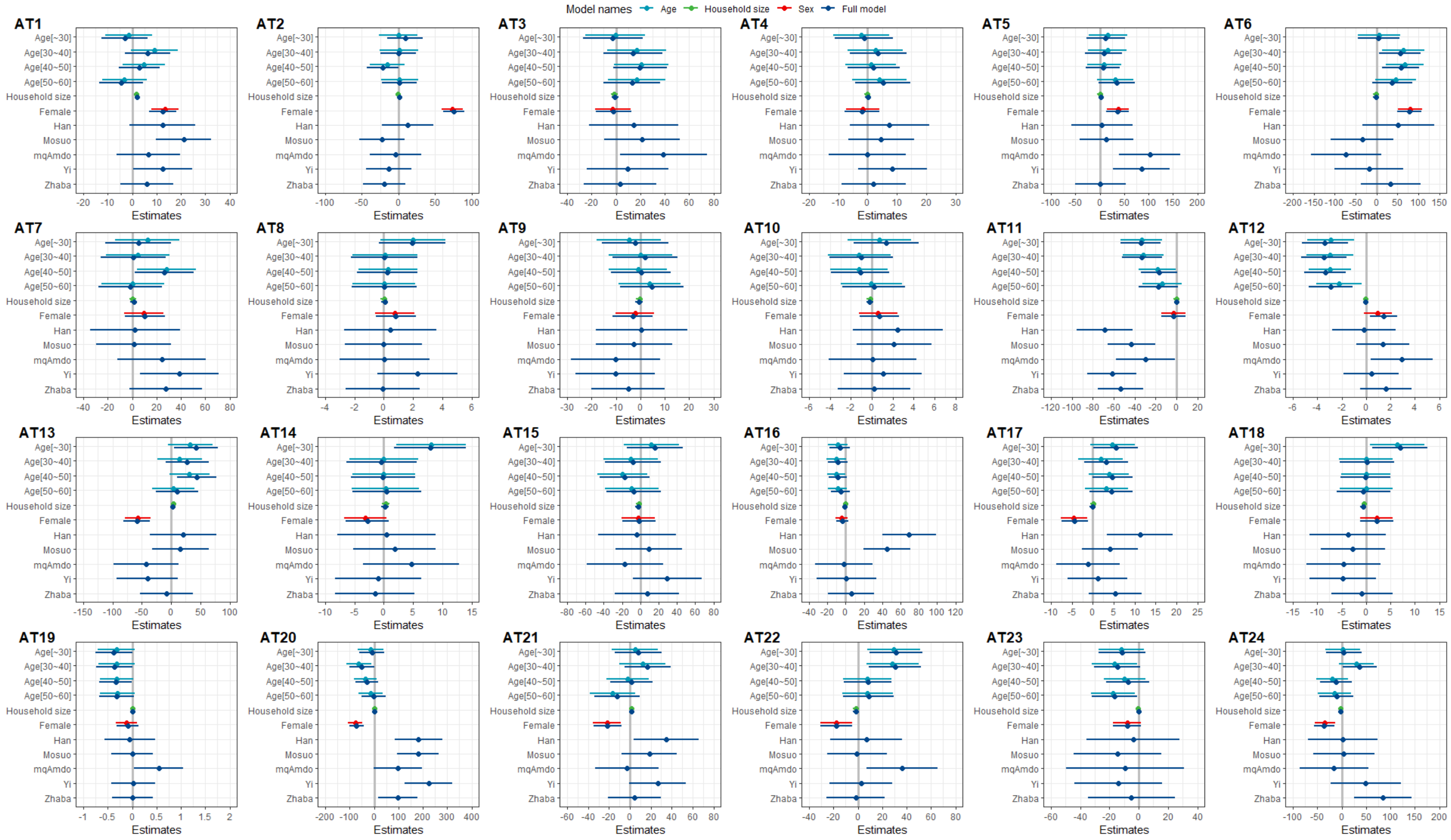

Multilevel models of predicting time spent on each activity controlling for age cohorts and household size, as well as ethnic groups as covariates, and Village ID as random effects (Taken from Chen 2023 FigureS4), related to STAR Methods. See Table S4 for specific activity codes.

Table S1. Best-fitting model estimates, related to Figure 1. Ethnic group was set as a random effect in this model.

Variables	Coefficients	t-value	p-value
**(Intercept)**	**185.15 (29.30)**	**6.32**	**0.0000**
**Age (ref: below30)**			
**Age (30,40)**	**9.52 (4.82)**	**1.97**	**0.0488**
Age (40,50)	4.33 (4.45)	0.97	0.3310
Age (50,60)	-6.23 (4.79)	-1.30	0.1943
**Age >60**	**-10.11 (4.80)**	**-2.10**	**0.0358**
BMI	-0.70 (0.37)	-1.92	0.0548
**Dispersal state (ref: nataler)**			
**Disperser**	**6.36 (3.11)**	**2.05**	**0.0412**
Physical state (ref: as usual)			
Being unwell or during lactation	-13.34 (9.96)	-1.34	0.1809
Ever removal of wristband (Ref: no)			
Ever removed	-6.37 (4.34)	-1.47	0.1428
**OSRv**	**-121.07 (55.69)**	**-2.17**	**0.0301**
**Sex (ref: female)**			
**Male**	**-102.80 (35.78)**	**-2.87**	**0.0042**
**OSRv X Sex (ref: female)**			
**OSRv: Male**	**178.35 (69.80)**	**2.56**	**0.0108**

Table S2. Model selection results with step gap between sexes per village as the dependent variable, related to Figure 4 and STAR Methods. Model 1: GLS model with fixed effects only. Model 2: Null mixed-effect model with the ethnic group as random effect only. Model 3: Control model with ethnic groups as a random effect. Model 4: Null mixed-effect model with OSR as a fixed effect, and ethnic groups as a random effect. Model 5: Global model.

Model name	Class	Df	logLik	AICc	Delta	weight
5	Lme	11	-5845.41	11713.26	0.00	0.87
4	Lme	4	-5854.49	11717.05	3.79	0.13
3	Lme	10	-5870.14	11760.65	47.39	0.00
2	Lme	3	-5877.31	11760.65	47.39	0.00
1	Gls	2	-5998.90	12001.82	288.56	0.00

Table S3. The variation coefficient of Miband2 and Fitbit Charge2 for each round in an experimental validation in the lab, related to STAR Methods. Miband2 and Fitbit Charge2 are two kinds of activity trackers. A, B, C, D indicate different subjects; T1, T2, T3, T4 indicate different period (the Time interval equals twenty minutes). Set 1: Six Fitbit Charge2 + four Miband2; Set 2: Another seven Fitbit Charge2 and another four Miband2. The coefficient of variation (CV) is defined as the ratio of the standard deviation to the mean. (Taken from Chen 2023 Table S1)

			Set 1				Set 2	
**Round**	**A-T1**	**A-T2**	**B-T3**	**B-T4**	**C-T1**	**C-T2**	**D-T3**	**D-T4**
Miband2								
	1278	1708	2062	2058	1635	1697	2085	2141
Steps	1205	1719	2082	2130	1600	1649	2056	2129
	1444	1614	2082	2114	1712	1680	2059	2145
	1486	1711	2084	2145	1621	1706	2086	2165
MEAN	1353.25	1688	2077.5	2111.75	1642	1683	2071.5	2145
SD	115.65	42.91	8.99	32.91	42.29	21.74	14.04	12.96
VAR	17832.92	2455.33	107.67	1444.25	2384.67	630	263	224
CV	0.09	0.03	0	0.02	0.03	0.01	0.01	0.01
Fitbit Charge2								
	1442	1738	1971	2022	1594	1722	1963	2024
	1431	1534	1971	1927	1591	1445	1962	1701
	1489	1654	2081	2030	1581	1731	1962	1971
	1495	1507	2080	1924	1603	1713	1965	1755
Steps	1469	1737	1968	2028	1585	1708	1958	1819
	1489	1507	2083	2039	1584	1711	1958	2051
	1385	1468	1971	1929				
MEAN	1457.14	1592.14	2017.86	1985.57	1589.67	1671.67	1961.33	1886.83
SD	37.27	106.49	54.99	51.24	7.39	101.66	2.56	135.01
VAR	1620.14	13229.14	3527.48	3062.95	65.47	12401.47	7.87	21872.97
CV	0.03	0.07	0.03	0.03	0	0.06	0	0.07

Table S4. Activity code and details, related to STAR Methods. (Taken from Chen 2023 Table S7).

A1 Housework of low intensity, e.g., watering the flowers, washing dishes, wiping the table, building a fire, and lighting bulbs. (LMET)

A2 Housework of moderate intensity, e.g., cooking a meal, sweeping the floor, mopping the floor, and washing clothes by hand. (MMET)

A3 Housework of high intensity, e.g., carrying heavy boxes or furniture, slaughtering poultry, sawing wood, and carpentry. (HMET)

A4 Farming of low intensity e.g., mechanized processing of corn. (LMET)

A5 Farming of moderate intensity (e.g., chasing cattle, milking, feeding livestock). (MMET)

A6 Farming of high intensity (press grass, baling hay, cleaning the barn, shoveling grain, watering when seeding, poultry work) (HMET)

A7 Digging herbs and fungi. (MMET)

A8 Tourism-related activities of low intensity (e.g., cashier). (LMET)

A9 Tourism-related activities of moderate intensity (e.g., being a waiter, washing dishes, serving dishes). (MMET)

A10 Tourism-related activities of high intensity (e.g., boating). (HMET)

A11 Religious activities of low intensity (quietly praying, washing dishes or sacrificial supplies, etc.). (LMET)

A12 Religious activity of moderate intensity (praising with dance or run, spiritual dancing or kowtowing). (MMET)

A13 Building/painting. (MMET)

A14 Sports, e.g., playing basketball. (MMET)

A15 Childcare. (MMET)

A16 Eating. (MMET)

A17 Self-care, e.g., taking a bath or brushing teeth. (MMET)

A18 Studying. (MMET)

A19 Teaching. (MMET)

A20 Entertainment, e.g., playing cards, watching TV, sitting, and chatting. (LMET)

A21 Purposeful traffic, e.g., walking marriage. Or drive a tractor, drive a harvester, and do something related to farm work. (MMET)

A22 Traffic no purpose reported, e.g., driving. (MMET)

A23 Sleeping. (LMET)

A24 Others.

Table S5. The relationship between time-budget data and step counts recorded by the accelerometer (N=561). The Iteration column is added indicating how many iterations were required to reach the stopping rule in the permutation test ‘ImPerm’ in R version 3.3.1. (Taken from Chen 2023 Table S2).

Activities	Estimate	Iterations	P-value
LI Housework	1.875	51	0.7451
MI Housework	5.331	51	1.0000
HI Housework	10.003	5000	0.0092**
LI Subsistence work	20.281	2112	0.0455*
MI Subsistence work	9.683	5000	<0.001***
HI Subsistence work	4.198	890	0.1011
Gathering work	14.898	5000	<0.001***
Serving work	-6.952	51	1.0000
LI Religious act	-8.190	311	0.2444
MI Religious act	82.404	212	0.3208
Building	1.564	51	1.0000
Sports	19.108	443	0.1851
Childcare	-9.111	5000	0.0030**
Eating	2.381	51	1.0000
Self-care	-37.660	5000	<0.001***
Leisure	-4.328	5000	0.0118*
Hanging around	1.086	158	0.3924
Driving	-2.102	51	1.0000
Height/cm	-89.439	3844	0.0255*
Weight/0.5kg	24.619	542	0.1568
